# Innate Immune Response to *Mycobacterium tuberculosis* Beijing and Other Genotypes

**DOI:** 10.1371/journal.pone.0013594

**Published:** 2010-10-25

**Authors:** Chongzhen Wang, Pascale Peyron, Olga Mestre, Gilla Kaplan, Dick van Soolingen, Qian Gao, Brigitte Gicquel, Olivier Neyrolles

**Affiliations:** 1 Centre National de la Recherche Scientifique, Institut de Pharmacologie et de Biologie Structurale, Toulouse, France; 2 Université de Toulouse, Université Paul Sabatier, Institut de Pharmacologie et de Biologie Structurale, Toulouse, France; 3 Unit of Mycobacterial Genetics, Institut Pasteur, Paris, France; 4 Laboratory of Mycobacterial Immunity and Pathogenesis, Public Health Research Institute Center at the University of Medicine and Dentistry of New Jersey, Newark, New Jersey, United States of America; 5 Tuberculosis Reference Laboratory, National Institute for Public Health and the Environment, Bilthoven, The Netherlands; 6 Departments of Pulmonary Diseases and Medical Microbiology, Radboud University, Nijmegen, The Netherlands; 7 Key Laboratory of Medical Molecular Virology, Shanghai Medical College, Fudan University, Shanghai, China; University of Hyderabad, India

## Abstract

**Background:**

As a species, *Mycobacterium tuberculosis* is more diverse than previously thought. In particular, the Beijing family of *M. tuberculosis* strains is spreading and evoluating throughout the world and this is giving rise to public health concerns. Genetic diversity within this family has recently been delineated further and a specific genotype, called Bmyc10, has been shown to represent over 60% of all Beijing clinical isolates in several parts of the world. How the host immune system senses and responds to various *M. tuberculosis* strains may profoundly influence clinical outcome and the relative epidemiological success of the different mycobacterial lineages. We hypothesised that the success of the Bmyc10 group may, at least in part, rely upon its ability to alter innate immune responses and the secretion of cytokines and chemokines by host phagocytes.

**Methodology/Principal Findings:**

We infected human macrophages and dendritic cells with a collection of genetically well-defined *M. tuberculosis* clinical isolates belonging to various mycobacterial families, including Beijing. We analyzed cytokine and chemokine secretion on a semi-global level using antibody arrays allowing the detection of sixty-five immunity-related soluble molecules. Our data indicate that Beijing strains induce significantly less interleukin (IL)-6, tumor necrosis factor (TNF), IL-10 and GRO-α than the H37Rv reference strain, a feature that is variously shared by other modern and ancient *M. tuberculosis* families and which constitutes a signature of the Beijing family as a whole. However, Beijing strains did not differ relative to each other in their ability to modulate cytokine secretion.

**Conclusions/Significance:**

Our results confirm and expand upon previous reports showing that *M. tuberculosis* Beijing strains in general are poor *in vitro* cytokine inducers in human phagocytes. The results suggest that the epidemiological success of the Beijing Bmyc10 is unlikely to rely upon any specific ability of this group of strains to impair anti-mycobacterial innate immunity.

## Introduction

*Mycobacterium tuberculosis* is the most frequent cause of tuberculosis (TB) in humans. According to the most recent WHO statistics, this bacterium is responsible for nearly 1.5 million deaths and 10 million new cases every year worldwide [1]. Improvement in the understanding of the pathogenesis of *M. tuberculosis* and its interaction with its human host will undoubtedly help in the development of novel drugs and vaccines to control TB. In recent years, it has become more apparent that *M. tuberculosis* is a more diverse species than previously thought [2]. Indeed, lineages of *M. tuberculosis* can be identified on the basis of genetic and genomic differences, including single nucleotide polymorphisms (SNPs) and large sequence polymorphisms [3], [4], [5], [6], [7], [8]. Different *M. tuberculosis* lineages are more prevalent in specific geographical regions, and have been accordingly named Indo-Oceanic, East Asian, East African-Indian, Euro-American, and West African lineages [2]. They have also been shown to be preferentially associated with specific human populations, suggesting adaptation to human host genotypes [7], [9], [10]. To which extend the immune response is adapted to these different lineages is starting to be explored. Such insights may provide significant help for developing novel intervention strategies.

The East Asian lineage, which predominantly includes the “Beijing” family of *M. tuberculosis* strains, is distributed mainly in east and southeast Asia, where it may account for 50% of all strains isolated in this region [11]. In recent years, Beijing strains have spread to many other parts of the world, including the US, Western Europe, and South Africa, and this accounts for over 13% of all *M. tuberculosis* strains worldwide [11], [12], [13]. Both immigrant and non-immigrant populations are affected and this raises a major public health concern. Clinical studies have suggested that patients infected with Beijing strains may be more prone disease progression [14], [15], more likely to have smear-positive sputa [16], have higher risk of developing extrapulmonary TB [17], and are less likely to be successfully treated [16], [18]. Taken together these observations suggest that Beijing strains of *M. tuberculosis* are hypervirulent. Studies in animal models have confirmed hypervirulence of Beijing strains as compared to other clinical isolates [11]. Other mechanisms that could account for the epidemiological success of Beijing strains include accumulation of drug resistance, adaptation to human immunity genes and genotypes, and the capacity to elicit different innate and adaptive immune responses compared with other *M. tuberculosis* strains (reviewed in [11]).

Cytokines and chemokines secreted by innate immune cells, such as macrophages (Mϕ) and dendritic cells (DC) play a crucial part in the host defense against *M. tuberculosis* [19], [20]. Differential induction of these molecules could contribute to variations in virulence, granuloma formation, and latency *versus* immunopathology and transmission. A number of studies in cells from mice and humans have concluded that, compared with reference strains, Beijing strains induce more type 1 interferon and less inflammatory cytokines, namely tumor necrosis factor (TNF), interleukin (IL)-1β, IL-12p40, IL-6 and IL-10. [21], [22], [23], [24], [25], [26], [27], [28], [29]. The most commonly used reference strains in these studies were H37Rv and CDC1551. The mechanisms underlying the characteristics of Beijing strains are not fully understood, and may involve the production of specific lipids [24], [30], [31].

In the present study, we further explored the innate immune response to the Beijing family of *M. tuberculosis* strains. The strains of this family have recently been clustered into over 20 genotypes/groups on the basis of SNPs identified in DNA replication, recombination and repair genes ([8]). One such group (named Bmyc10) appears to be largely over-represented, and represents over 60% of all Beijing strains that have been analyzed, regardless of their geographical origin. Another Beijing group, Bmyc25, represents 9% of the Beijing strains analyzed and includes the Gran Canaria outbreak strain GC1237 [32]. The clear recent expansion of the Bmyc10 group, and, to a lesser extend, the Bmyc25 group, as compared with ancestral Beijing groups (Table S1), suggests that these subfamilies may be more adapted to their human hosts. We hypothesized that the Bmyc10 and possibly Bmyc25 strains might differ from other ancestral and minor Beijing subfamilies in their ability to stimulate innate immune cells and induce Mϕ and DC cytokine/chemokine secretion. In our investigations, we used a combination of semi-global protein array- and ELISA-based assays, as well as an *in vitro* model of granuloma formation. Among the 65 cytokines and other immune mediators that were analyzed, we did not find a single molecule that was differentially modulated by Bmyc10 or Bmyc25 strains, compared with other Beijing strains. However, we confirmed that low production of TNF, IL-6 and IL-10 by infected human Mϕ and DC *in vitro* is a signature of the Beijing family as a whole. We also report that Beijing strains consistently induce less GRO-α, as compared with H37Rv and other *M. tuberculosis* lineages.

## Results and Discussion

**Figure 1:**
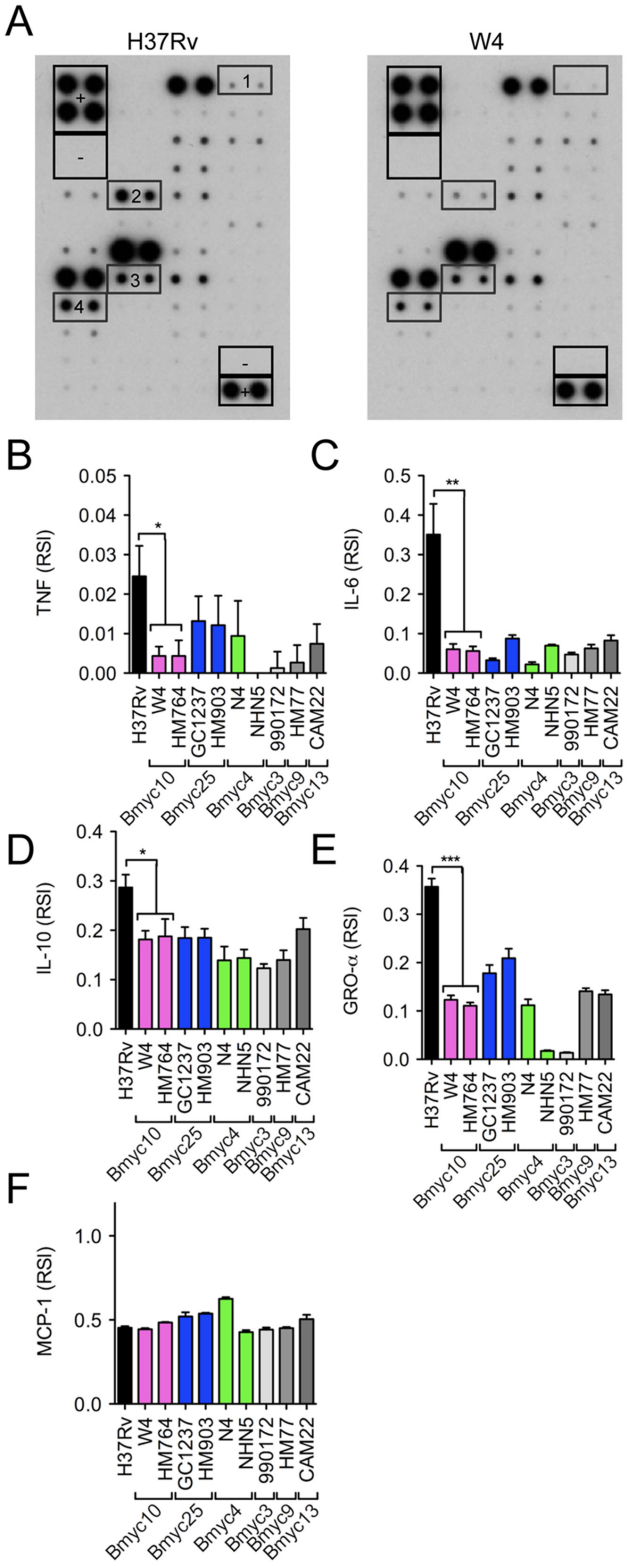
Semi-global protein analysis reveals differences in the cytokine/chemokine profile of Mϕ infected with either *M. tuberculosis* H37Rv or the Beijing strains. Human Mϕ were infected with either *M. tuberculosis* H37Rv or representive Beijing strains for 18 h and the cell culture supernatants were analyzed for cytokine/chemokine content using Human Cytokine Antibody Array 3. The detection signals were captured with X-ray films. A) Representative membrane scanning analysis of supernatants from cells infected with H37Rv (left panel) and the W4 (Beijing, Bmyc10 group, right panel) strains. +, positive control spots; −, negative control spots; 1, TNF spots; 2, IL-6 spots; 3, IL-10 spots; 4, GRO-α spots. B–E) The signals on the membranes were quantified by densitometry. Results obtained for TNF (B), IL-6 (C), IL-10 (D), GRO-α (E) and MCP-1 (F) are shown and data represent means±s.d. of relative signal intensity of the duplicate spots. Data were analyzed using the Student's t-test. *, *P*<0.05; **, *P*<0.01; ***, *P*<0.001. The complete list of cytokine/chemokines subjected to detection by the array is presented in Table S2.

**Figure 2:**
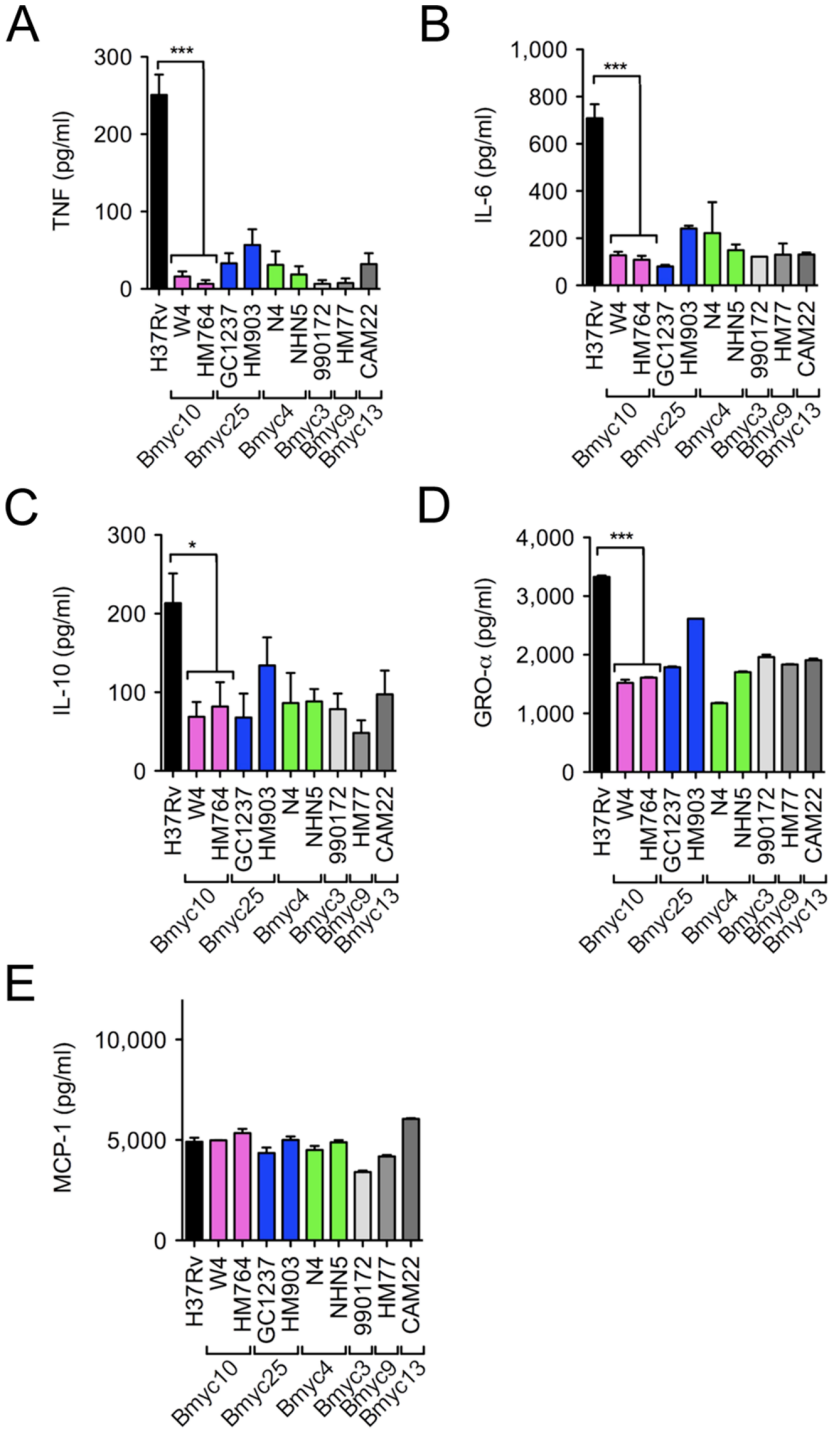
ELISA confirmation of differences in IL-6, IL-10, GRO-α, and TNF secreted by Mϕ upon infection with either *M. tuberculosis* H37Rv or Beijing strains. Human Mϕ were infected with *M. tuberculosis* H37Rv or representive Beijing strains for 18 h and the cell culture supernatants were analyzed for cytokine/chemokine content using dedicated ELISA kits. Data represent the means±s.d. of TNF (A), IL-6 (B), IL-10 (C), GRO-α (D), and MCP-1 (E) concentration in four samples (n = 4) from one representative donor out of four independent donors. Data were analyzed using the Student's t-test. *, *P*<0.05; **, *P*<0.01; ***, *P*<0.001.

Based on the phylogenetic tree of the Beijing family that has been recently constructed by Mestre *et al.* [33], a total of nine representative strains were selected from the different subfamilies. These included two strains from the over-represented modern groups Bmyc10 and Bmyc25, two strains from the ancestral group Bmyc4, and three strains from three minor groups, namely Bmyc3, Bmyc9 and Bmyc13 (Table S1). Human monocytes were collected from healthy blood donors and differentiated into Mϕ in the presence of monocyte-colony stimulating factor (M-CSF). Mϕ were infected with the different strains at a multiplicity of infection (MOI) of 5 bacteria per cell. At 4 h and 18 h following infection, the culture medium was collected and analyzed for cytokine/chemokine content using dedicated antibody arrays, which could assay a total of 65 cytokines, chemokines, growth factors and other soluble immune mediators. The signals were captured with X-ray films (Figure 1A), and quantified by densitometry (Figure 1B–F). Thirty-four molecules were detected at varying levels in the supernatants of infected cells at 4 h and 18 h post-infection (Table 1& Table S2). Production of IL-6, IL-10 and GRO-α by Mϕ at 18 h after infection was significantly less for Beijing strains than for H37Rv (Figure 1C–E). The production of other molecules, such as monocyte chemotactic protein (MCP)-1 (Figure 1F) did not differ between H37Rv and Beijing strains, indicating that cells had been infected at comparable MOI. We also confirmed that, as previously reported, Beijing strains induce less TNF than H37Rv in Mϕ after 18 h infection (Figure 1B), although this was barely visible in the protein arrays (Figure 1A). All these results were confirmed by ELISA in two subsequent independent experiments (Figure 2). These findings suggest that, as previously reported, Beijing strains induce less TNF, IL-6 and IL-10 *in vitro* in Mϕ than H37Rv. In addition we found that Beijing strains also induce less GRO-α than the reference strain, and this has not been previously reported. No differences were observed among the Beijing strains, except minor variations, notably GRO-α production (Figure 1E), which were not confirmed by ELISA quantification.

**Table 1:** Cytokines, chemokines and growth factors secreted by H37Rv- and W4 (Beijing Bmyc10)-infected human monocyte-derived macrophages^1^.

	Infection time			
	4 h		18 h	
	Strain		Strain	
Cytokine	H37Rv	W4	H37Rv	W4
IL-2	ND	ND	+/−	+/−
IL-3	+/−	+/−	+/−	+/−
IL-6	+/−	+/−	+	+/−
IL-7	+/−	+/−	+/−	+/−
IL-8	++	++	++	++
IL-10	+/−	+/−	+	+
TNF-α	+	+	+/−	ND
I-309/CCL1	ND	ND	+/−	+/−
MCP-1/CCL2	+	+	++	++
MIP-1 α/CCL3	+	+	+	+
MIP-1 β/CCL4	++	++	++	++
RANTES/CCL5	+/−	+/−	+	+
MCP-3/CCL7	ND	ND	+/−	+/−
MCP-2/CCL8	ND	ND	+/−	+/−
MIP-1 δ/CCL15	ND	ND	+/−	+/−
MIP-3 α/CCL20	ND	ND	+/−	+/−
MDC/CCL22	+/−	+/−	+/−	+/−
Eotaxin-2/CCL24	ND	ND	+/−	+/−
Eotaxin-3/CCL26	+/−	+/−	+/−	+/−
GRO	++	++	++	++
GRO-α/CXCL1	+/−	+/−	+	+
ENA-78/CXCL5	ND	ND	+/−	+/−
GCP-2/CXCL6	+/−	+/−	+/−	+/−
NAP-2/CXCL7	+/−	+/−	+/−	+/−
IP-10/CXCL10	+/−	+/−	+/−	+/−
I-TAC/CXCL11	+/−	+/−	+/−	+/−
CXCL16	ND	ND	+/−	+/−
Fractalkine/CX3CL1	+/−	+/−	+/−	+/−
Lymphotactin/XCL1	ND	ND	+/−	+/−
SCF/KITLG	ND	ND	+/−	+/−
MCSF/CSF-1	+/−	+/−	+/−	+/−
GM-CSF/CSF-2	+/−	+/−	+/−	+/−
EGF	+/−	+/−	+/−	+/−
Oncostatin M	ND	ND	+/−	+/−

1 Symbols indicate relative signal intensity (RSI) in the membrane array. +/−, weak production; 0.01<RSI<0.1; +, 0.1<RSI<0.5; ++, strong production, RSI>0.5; ND, not detected (RSI<0.01).

Human monocyte-derived DC generated in the presence of granulocyte/monocyte colony-stimulating factor (GM-CSF) and IL-4 were used in similar experiments. The complete set of data is shown in Table S3. The results for DC were similar with lower production of TNF and IL-6 following infection with Beijing strains compared to H37Rv (Table S3 & Figure S1). GRO-α was not detected in the supernatant of infected DC but slight differences between H37Rv- and Beijing-infected cell supernatants were observed for the “GRO” spot on the membranes, which suggest that cells may differentially secrete either GRO-β/MIP-2α/CXCL2 or GRO-γ/MIP-2β/CXCL3 or both. In addition, MIP-1α secretion was diminished in DC infected with the Beijing strains as compared to with H37Rv (Table S3), which was confirmed by ELISA (Figure S1). No differences in IL-10 production were observed (data not shown).

These results confirm and expand upon previous studies, and show that the Beijing strains of *M. tuberculosis* are weak cytokine producers compared to the H37Rv reference strain, at least regarding TNF, IL-6, IL-10 and GRO-α.

**Figure 3:**
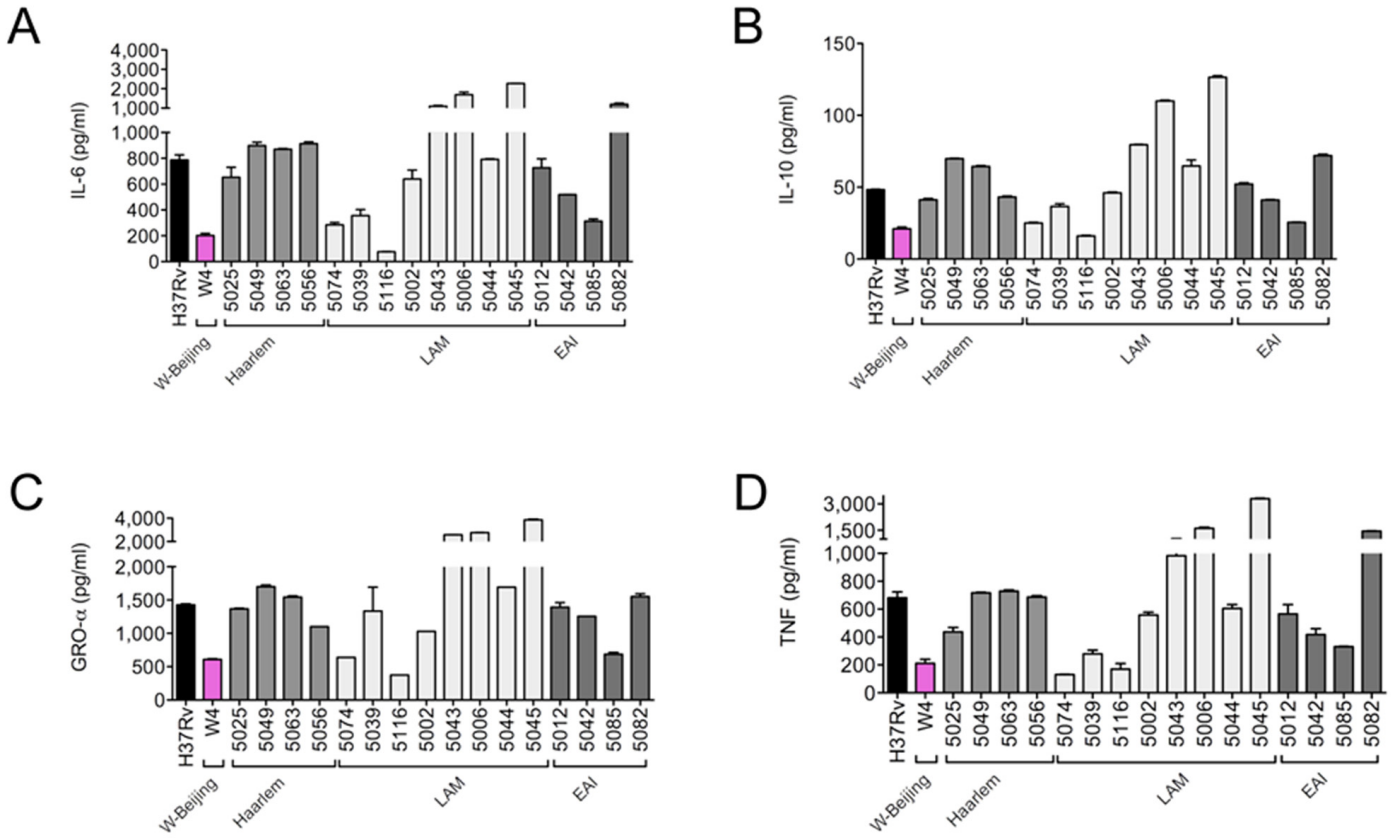
Cytokine secretion profiles of Mϕ infected with various major *M. tuberculosis* lineages. Human Mϕ were infected with either *M. tuberculosis* H37Rv or representive strains from the Beijing, Haarlem, LAM and EAI families for 18 h and the cell culture supernatants were analyzed for cytokine/chemokine content using dedicated ELISA kits. Data represent the means±s.d. of IL-6 (A), IL-10 (B), GRO-α (C), and TNF (D) concentration in four samples (n = 4) from one representative donor out of two independent donors.

We next assessed whether such a signature was specific to the Beijing family or was common to other *M. tuberculosis* lineages. The Euro-American lineage, to which H37Rv belongs, and the East-Asian lineage (Beijing) are both considered as “modern” *M. tuberculosis* lineages, lacking in particular the TbD1 genomic region [7], [34]. We wanted to extend our investigations to other “modern” *M. tuberculosis* families (Haarlem and LAM families) and to an “ancient” family (the EAI family, belonging to the Indo-Oceanic lineage). We infected human Mϕ with 16 strains from these families (Table S1), in addition to H37Rv and Beijing strains, and compared the cytokine/chemokine production profiles. As shown in Figure 3, we found a clear heterogeneity among the non-Beijing strains with respect to cytokine/chemokine secretion. The Haarlem and most of the LAM strains induced similar cytokine levels to H37Rv, consistent with these strains belonging to the Euro-American lineage. However two of the LAM strains induced cytokine levels that were closer to those observed with the Beijing strains. A strong heterogeneity was also observed in the ancient EAI strains, with some strains inducing H37Rv-like profiles of cytokine secretion, and other inducing Beijing-like profiles (Figure 3). As a whole, about two thirds of the non-Beijing strains induced higher cytokine/chemokine levels than the Beijing strains, while the others induced comparable cytokine/chemokine levels. Thus, among the different lineages of *M. tuberculosis*, the Beijing strains are homogeneous low-cytokine/chemokine inducers.

**Figure 4:**
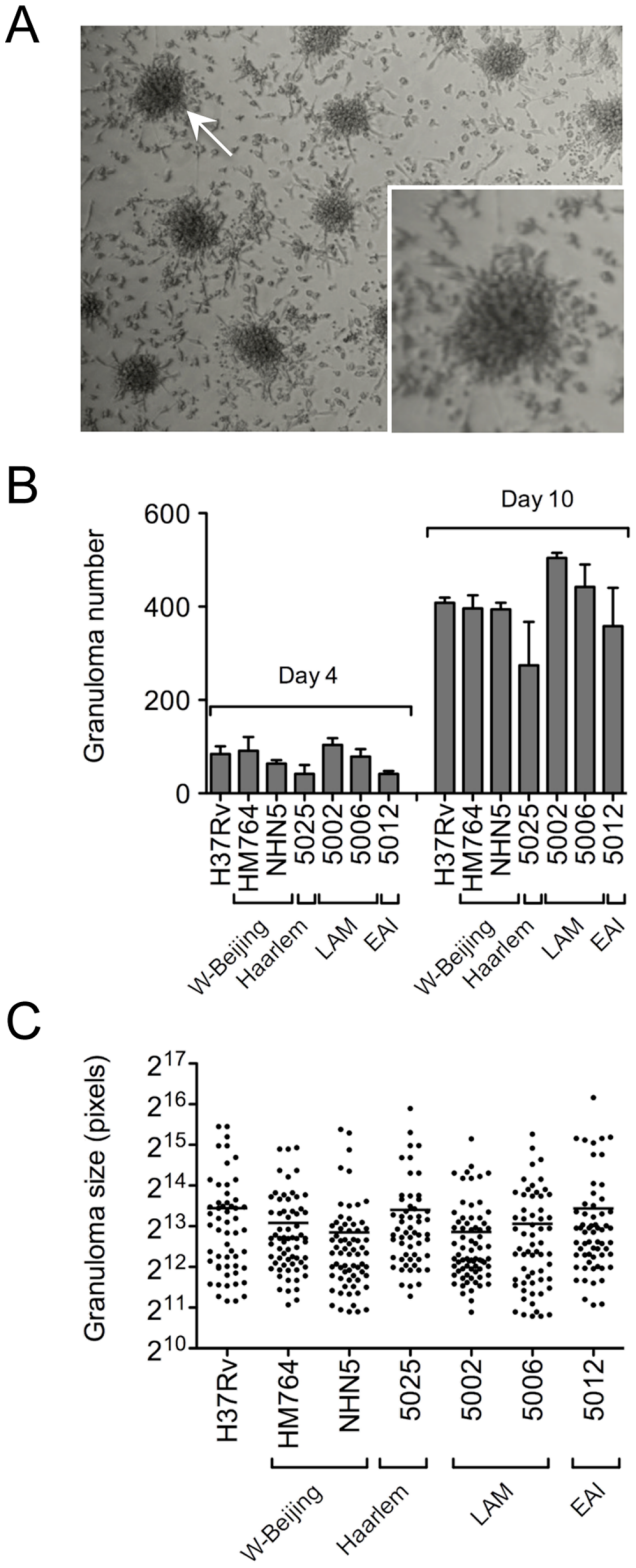
*In vitro* granuloma formation upon PBMC infection with various major *M. tuberculosis* lineages. Human whole PBMC were infected with either *M. tuberculosis* H37Rv or representive strains from the Beijing, Haarlem, LAM and EAI families for 4 or 10 days and monitored for granuloma formation as observed under the light microscope. A) Typical granulomas observed 10 days after infection; the white arrow indicates a granuloma shown at higher magnification in the inset. B) Data represent the means±s.d. number of granuloma at 4 and 10 days, scored in two independent observation fields. C) Data represent the means±s.d. granuloma size 10 days after infection; between 38 and 55 granulomas were recorded in two independent observation fields. Data were analyzed using the Student's t-test; no significant differences were observed among the different conditions.

Finally, because some of the differentially induced immune mediators, such as TNF, are involved in granuloma formation, we assessed whether differential cytokine secretion correlates with *in vitro* granuloma formation. Following a previously described procedure [35], we infected total peripheral blood mononuclear cells (PBMC) with different Beijing and non-Beijing strains at a MOI of 1 bacterium/2,000 cells, and monitored granuloma formation (Figure 4A) over time. The number (Figure 4B) and size (Figure 4C) of granulomas induced by the different strains was found to be similar.

Taken together, our results show that following infection of human Mϕ and DC, the Beijing group Bmyc10 is similar to the Bmyc4 ancestral group and the other minor groups in terms of cytokine/chemokine production and *in vitro* granuloma formation, at least at the early time-points examined here; we cannot exclude that longer infection times may have revealed differences in production of other specific cytokines (e.g. IL-12). The Bmyc10 subfamily represents over 60% of the Beijing clinical isolates in several parts of the world. This suggests that this subfamily may have an evolutionary advantage compared with the other Beijing groups. Our data indicate that this advantage does not depend upon a differential ability of Bmyc10 strains to impair production of cytokines and other immune mediators in innate immune cells as opposed to other Beijing groups. However, we cannot conclude that the *in vivo* immune response to Bmyc10 strains is the same as that to other Beijing. This indicates the need for further investigations using animal models of TB. Another possibility is that specific mutations in DNA replication, recombination and repair genes, such as the Bmyc10-specific Gly^58^∏Arg mutation in the *mutT2* gene, have conferred a selective advantage to these strains by leading to transient mutator phenotypes resulting in strain evolution [33].

We also report that Beijing strains, regardless of subfamily, commonly induce lower levels of TNF, IL-6, IL-10 and GRO-α compared with H37Rv strain. Experiments including additional strains outside the Beijing family showed that the low cytokine-production is a uniform signature of this family. These results were found in human monocyte-derived Mϕ and DC, and confirm and expand upon previous reports that used other *in vitro* and *ex vivo* cell model systems not including monocyte-derived Mϕ and DC [23], [24], [25], [26], [27], [28], [29]. The mechanisms responsible for low cytokine production by Beijing strains remain to be identified; we can anticipate that it is unlikely to rely solely on the production of phenolic glycolipid (PGL) because we could not establish a direct relationship between PGL production (e.g. in the W4 strain [30]) or absence of PGL (e.g. in the N4 and NHN5 strains [30]), and a differential ability of these strains to impair cytokine production.

The inflammatory response of Mϕ to *M. tuberculosis* promotes local inflammation and innate control of bacterial infection by Mϕ, neutrophils and natural killer cells [36], [37]. It also promotes migration of antigen-specific T cells from the draining lymph node to the site of *M. tuberculosis* infection where they exert adaptive control of *M. tuberculosis* through granuloma formation [38]. Of the 4 cytokines and chemokines that were induced at lower levels by the Beijing-infected Mϕ, TNF, IL-6 and GRO-α are proinflammatory [36], while IL-10 could play a regulatory role in preventing immunophothology [36], [39]. Lower induction of these cytokines by the Beijing strains may therefore lead to either a higher local inflammation or to a weaker innate and adaptive control of mycobacteria resulting in a hypervirulent *M. tuberculosis* phenotype. Further exploration, including *in vivo* investigations, is required to determine the net effect of these multiple modulations in cytokine secretion. The finding that GRO-α/CXCL1 and MIP-1α/CCL3 secretion is impaired in Beijing-infected cells is novel. These chemokines are chemotactic for granulocytes, including neutrophils. Our results suggest that neutrophil recruitment may be impaired or delayed in the lungs of Beijing-infected hosts, which should also be further examined *in vivo*.

## Materials and Methods

### Mycobacteria strains and culture medium

The strains of *M. tuberculosis* that were used in this study are listed in Table S1. Bacilli were cultured in Middlebrook 7H9 broth (BD-Diagnostic Systems) supplemented with 10% Middlebrook ADC enrichment medium (BD-Diagnostic Systems) and 0.05% Tween-80 (Sigma-Aldrich). The H37Rv strain used here was obtained from S. Cole (Institut Pasteur, Paris) and was the one used in the initial *M. tuberculosis* genome-sequencing project. After a limited number of *in vitro* expansion passages, bacteria have been aliquoted and frozen. Aliquots were thawed and cultured 24 h before infection, in order to minimize loss of virulence.

### Preparation of monocyte derived macrophages and dendritic cells, and cell infection

Monocytes were obtained from healthy blood donors (Etablissement Français du Sang, EFS, Toulouse). Written informed consents were obtained from the donors under EFS contract n°21/PVNT/TOU/IPBS01/2009-0052. Following articles L1243-4 and R1243-61 of the French Public Health Code, the contract was approved by the French Ministry of Science and Technology (agreement n°AC 2009-921).

Monocytes were differentiated into Mϕ or DC following a previously published procedure [40]. Briefly, mononuclear cells were isolated from blood using Ficoll-Paque Plus (GE Healthcare). Monocytes were then purified using CD14 microbeads and MACS separation columns (Miltenyi Biotec). Monocytes were resuspended in RPMI-1640 medium (Invitrogen) and allowed to attach to 12-well polystyrene plates (Corning) for one hour at 37°C (700,000 cells/well). After washing with PBS, monocytes were cultured in RPMI-1640 medium supplemented with 10% heat-activated human serum (Sigma-Aldrich), 1% penicillin-streptomycin (Invitrogen), and 20 ng/ml M-CSF (Miltenyi Biotec) at 37°C under a 5% CO_2_ humidified atmosphere. On day 3, the culture was fed with complete medium containing a full dose of M-CSF. On day 5, the culture medium was changed to macrophage serum-free medium (Invitrogen) supplemented with 20 ng/ml M-CSF. On day 6, the monocyte-derived Mϕ were ready for infection. DC were differentiated following a similar procedure where M-CSF was replaced by GM-CSF (10 ng/ml) and IL-4 (20 ng/ml).

Exponentially growing mycobacteria were centrifuged and resuspended in macrophage serum-free medium. Clumps were disassociated by 20 passages through a 26-G needle. An OD_600_ value between 0.2 and 1 was measured in a dilution of the bacterial suspension. The bacterial density in the suspension was estimated using the equation: bacteria/ml = OD_600_x10^8^xdilution. Infection of the cells was performed at a multiplicity of infection (MOI) of 5 bacteria/cell by changing the cell culture medium to a mycobacteria suspension in 1 ml macrophage serum-free medium. After 4 hours of incubation at 37°C, the culture medium was collected and sterilized by filtration. The cells were fed with fresh serum-free medium and cultured for an additional 14 h. The culture medium was again collected and sterilized. For *in vitro* granuloma formation, total PBMC were infected with the *M. tuberculosis* strains at a MOI of 1 bacteria/2,000 cells and were observed at different times after infection using a light microscope, as previously described [35].

### Semi-quantitative cytokine/chemokine antibody array assay and quantitative ELISA

Cytokines, chemokines and other growth factors and immune mediators present in the cell culture supernatants were assayed using Human Cytokine Antibody Array 3 and Human Chemokine Antibody Array 1 (RayBiotech). The individual signals were detected on Amersham Hyperfilm ECL (GE Healthcare) and were quantified using a GS-800 calibrated densitometer (Bio-Rad). The positive control signal on each array was used for normalization, according to the manufacturer's instructions.

ELISA kits were purchased from BD Biosciences (TNF, IL-6, IL-10, MCP-1) and R&D Systems (MIP-1α, GRO-α), and were used for cytokine/chemokine quantification according to the manufacturer's recommendations.

## Supporting Information

Figure S1 ELISA confirmation of differences in TNF, IL-6, and MIP-1α secreted by DC upon infection with either M. tuberculosis H37Rv or Beijing strains. Human DC were infected with *M. tuberculosis* H37Rv or representive Beijing strains for 18 h and the cell culture supernatants were analyzed for cytokine/chemokine content using dedicated ELISA kits. Data represent the means±s.d. of TNF (A), IL-6 (B), and MIP-1α (C) concentration in four samples (n = 4) from one representative donor out of two independent donors. Data were analyzed using the Student t-test. **, P<0.01; ***, P<0.001.
(1.46 MB TIF)

Table S1 *M. tuberculosis* strains used in the study.
(0.05 MB DOC)

Table S2 Cytokines, chemokines and growth factors secreted by H37Rv- and W4 (Beijing Bmyc10)-infected human monocyte-derived macrophages.
(0.13 MB DOC)

Table S3 Cytokines, chemokines and growth factors secreted by H37Rv- and W4 (Beijing Bmyc10)-infected human monocyte-derived dendritic cells.
(0.13 MB DOC)
