# Supplementary figures and images for: Innate Immune Response to Mycobacterium tuberculosis Beijing and Other Genotypes
Source: PLoS One. 2010 Oct 25;5(10):e13594. doi: 10.1371/journal.pone.0013594 (PMC2963601; doi:10.1371/journal.pone.0013594)

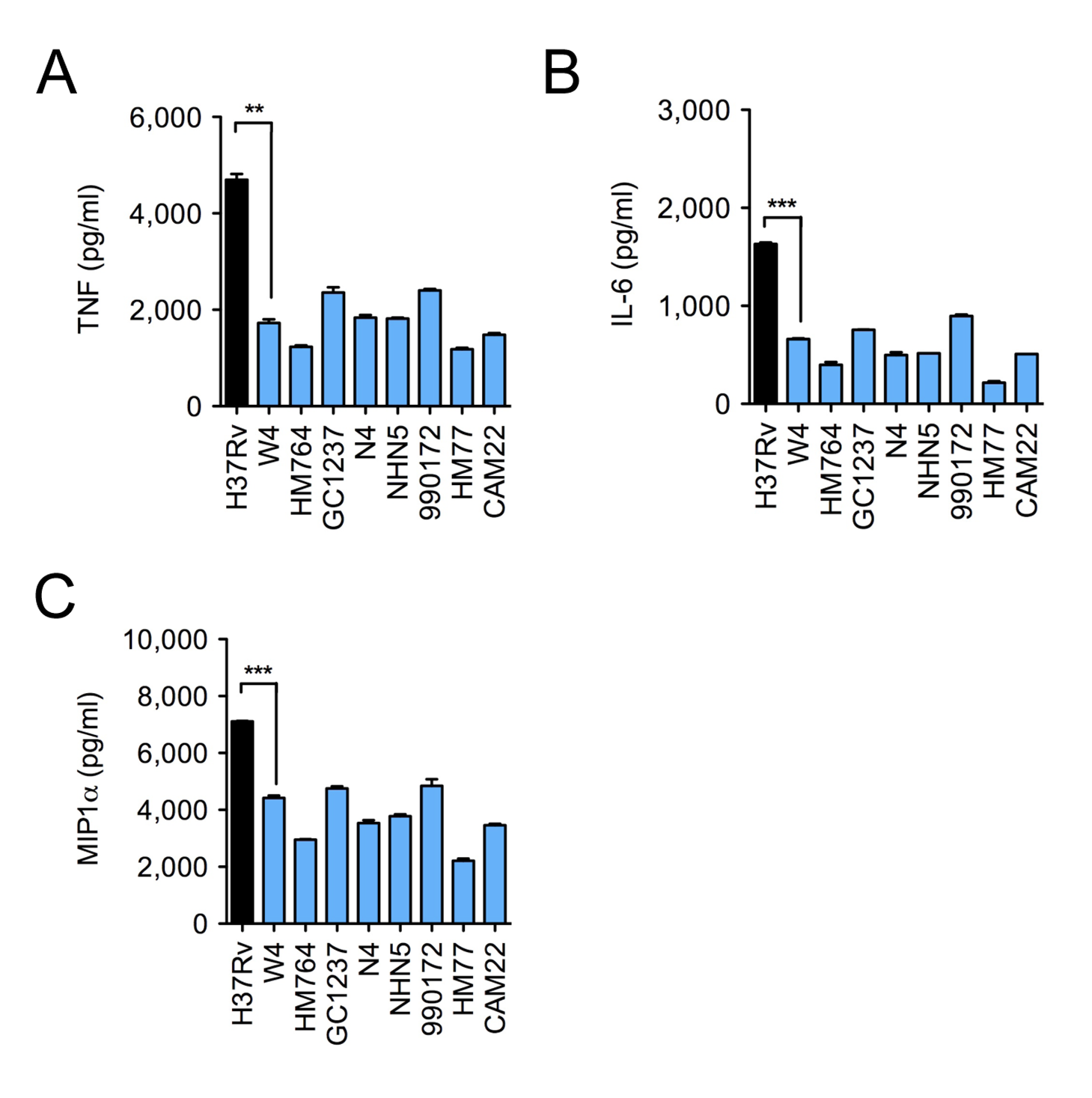

Supplement: Figure S1 — ELISA confirmation of differences in TNF, IL-6, and MIP-1α secreted by DC upon infection with either M. tuberculosis H37Rv or Beijing strains. Human DC were infected with M. tuberculosis H37Rv or representive Beijing strains for 18 h and the cell culture supernatants were analyzed for cytokine/chemokine content using dedicated ELISA kits. Data represent the means±s.d. of TNF (A), IL-6 (B), and MIP-1α (C) concentration in four samples (n = 4) from one representative donor out of two independent donors. Data were analyzed using the Student t-test. **, P<0.01; ***, P<0.001. (1.46 MB TIF) [file pone.0013594.s001.tif]
