# Supplementary material for: Innate Immune Response to Mycobacterium tuberculosis Beijing and Other Genotypes
Source: PLoS One. 2010 Oct 25;5(10):e13594. doi: 10.1371/journal.pone.0013594 (PMC2963601; doi:10.1371/journal.pone.0013594)
Supplement: Table S1 — M. tuberculosis strains used in the study. (0.05 MB DOC) [file pone.0013594.s002.doc]

**Table S1. *M. tuberculosis* strains used in the study^1^**

| **Strains** | **Genotype information** |
| --- | --- |
| H37Rv | Euro-American lineage |
| W4 | East Asian (Beijing) lineage, group Bmyc10 |
| HM764 | East Asian (Beijing) lineage, group Bmyc10 |
| CAM22 | East Asian (Beijing) lineage, group Bmyc13 |
| HM77 | East Asian (Beijing) lineage, group Bmyc9 |
| HM903 | East Asian (Beijing) lineage, group Bmyc25 |
| GC1237 | East Asian (Beijing) lineage, group Bmyc25 |
| NHN5 | East Asian (Beijing) lineage, group Bmyc4 |
| N4 | East Asian (Beijing) lineage, group Bmyc4 |
| 99-0172 | East Asian (Beijing) lineage, group Bmyc3 |
| 5025 | Euro-American lineage, H3 family |
| 5049 | Euro-American lineage, H3 family |
| 5063 | Euro-American lineage, H3 family |
| 5056 | Euro-American lineage, H3 family |
| 5039 | Euro-American lineage, LAM1 family |
| 5116 | Euro-American lineage, LAM1 family |
| 5002 | Euro-American lineage, LAM3 family |
| 5043 | Euro-American lineage, LAM3 family |
| 5006 | Euro-American lineage, LAM9 family |
| 5044 | Euro-American lineage, LAM9 family |
| 5045 | Euro-American lineage, LAM9 family |
| 5074 | Euro-American lineage, LAM10 family |
| 5012 | Indo-Oceanic lineage, EAI1 family |
| 5042 | Indo-Oceanic lineage, EAI1 family |
| 5085 | Indo-Oceanic lineage, EAI1 family |
| 5082 | Indo-Oceanic lineage, EAI5 family |

^1^Strains are classified according to their spoligotype as defined in

the SpolDB4 database [41].
