# Supplementary material for: Innate Immune Response to Mycobacterium tuberculosis Beijing and Other Genotypes
Source: PLoS One. 2010 Oct 25;5(10):e13594. doi: 10.1371/journal.pone.0013594 (PMC2963601; doi:10.1371/journal.pone.0013594)
Supplement: Table S3 — Cytokines, chemokines and growth factors secreted by H37Rv- and W4 (Beijing Bmyc10)-infected human monocyte-derived dendritic cells. (0.13 MB DOC) [file pone.0013594.s004.doc]

**Table S3.** Cytokines, chemokines and growth factors secreted by H37Rv- and W4 (Beijing Bmyc10)-infected human monocyte-derived dendritic cells^1^

|  | **Infection time** | | | |
| --- | --- | --- | --- | --- |
|  | **4 h** | | **18 h** | |
|  | **Strain** | | **Strain** | |
| **Cytokine** | **H37Rv** | **W4** | **H37Rv** | **W4** |
| IL-1α | ND | ND | ND | ND |
| IL-1β | ND | ND | ND | ND |
| IL-2 | ND | ND | ND | ND |
| IL-3 | +/- | +/- | ND | ND |
| IL-4 | ND | ND | ND | ND |
| IL-5 | ND | ND | ND | ND |
| IL-6 | +/- | +/- | ++ | + |
| IL-7 | ND | ND | +/- | +/- |
| IL-8 | ++ | ++ | ++ | ++ |
| IL-10 | +/- | +/- | +/- | +/- |
| IL-12 (p40p70) | ND | ND | ND | ND |
| IL-13 | ND | ND | ND | ND |
| IL-15 | ND | ND | ND | ND |
| IFN-γ | ND | ND | ND | ND |
| TGF-β1 | ND | ND | ND | ND |
| TNF-α | + | + | + | +/- |
| TNF-β | ND | ND | ND | ND |
| I-309/CCL1 | ND | ND | ND | ND |
| MCP-1/CCL2 | + | + | + | + |
| MIP-1 α/CCL3 | + | +/- | + | +/- |
| MIP-1 β/CCL4 | ++ | ++ | ++ | ++ |
| RANTES/CCL5 | +/- | +/- | +/- | +/- |
| MCP-3/CCL7 | ND | ND | ND | ND |
| MCP-2/CCL8 | ND | ND | ND | ND |
| Eotaxin/CCL11 | ND | ND | ND | ND |
| MCP-4/CCL13 | +/- | +/- | +/- | +/- |
| MIP-1 δ/CCL15 | ND | ND | +/- | +/- |
| HCC-4/CCL16 | ND | ND | ND | ND |
| TARC/CCL17 | ND | ND | ND | ND |
| PARC/CCL18 | ND | ND | ND | ND |
| MIP-3 β/CCL19 | ND | ND | ND | ND |
| MIP-3 α/CCL20 | ND | ND | ND | ND |
| MDC/CCL22 | +/- | +/- | + | + |
| MPIF-1/CCL23 | ND | ND | ND | ND |
| Eotaxin-2/CCL24 | + | + | + | + |
| TECK/CCL25 | ND | ND | ND | ND |
| Eotaxin-3/CCL26 | +/- | +/- | +/- | +/- |
| CTACK/CCL27 | ND | ND | ND | ND |
| CCL28 | ND | ND | ND | ND |
| GRO | +/- | +/- | ++ | + |
| GRO-α/CXCL1 | ND | ND | ND | ND |
| ENA-78/CXCL5 | ND | ND | ND | ND |
| GCP-2/CXCL6 | ND | ND | ND | ND |
| NAP-2/CXCL7 | +/- | +/- | +/- | +/- |
| MIG/CXCL9 | ND | ND | +/- | +/- |
| IP-10/CXCL10 | +/- | +/- | +/- | +/- |
| I-TAC/CXCL11 | +/- | +/- | +/- | +/- |
| SDF-1 α /CXCL12 | ND | ND | ND | ND |
| SDF-1 β /CXCL12 | ND | ND | ND | ND |
| BLC/CXCL13 | ND | ND | ND | ND |
| CXCL16 | +/- | +/- | +/- | +/- |
| Fractalkine/CX3CL1 | +/- | +/- | +/- | +/- |
| Lymphotactin/XCL1 | ND | ND | ND | ND |
| SCF/KITLG | ND | ND | ND | ND |
| MCSF/CSF-1 | ND | ND | +/- | +/- |
| GM-CSF/CSF-2 | ND | ND | +/- | +/- |
| GCSF/CSF-3 | ND | ND | ND | ND |
| EGF | ND | ND | +/- | +/- |
| IGF-I | ND | ND | ND | ND |
| Angiogenin | ND | ND | ND | ND |
| Oncostatin M | +/- | +/- | +/- | +/- |
| Thrombopoietin | ND | ND | ND | ND |
| VEGF | ND | ND | ND | ND |
| PDGF-β | ND | ND | ND | ND |
| Leptin | ND | ND | ND | ND |

^1^Symbols indicate relative signal intensity (RSI) in the membrane array. +/-, weak production ; 0.01<RSI<0.1; +, 0.1<RSI<0.5 ; ++, strong production, RSI>0.5 ; ND, not detected (RSI<0.01).
